# Preparation and characterization of superporous hydrogels as gastroretentive drug delivery system for rosiglitazone maleate

**Published:** 2010

**Authors:** N. Vishal Gupta, H.G. Shivakumar

**Affiliations:** Department of Pharmaceutics, JSS College of Pharmacy, JSS University, Karnataka, India

**Keywords:** Chitosan, Swelling, Gastric retention

## Abstract

**Background and the purpose of the study:**

Many drugs which have narrow therapeutic window and are absorbed mainly in stomach have been developed as gastroretentive delivery system. Rosiglitazone maleate, an anti-diabetic, is highly unstable at basic pH and is extensively absorbed from the stomach. Hence there is a need to develop a gastroretentive system. In this study a superporous hydrogel was developed as a gastroretentive drug delivery system.

**Methods:**

Chitosan/poly(vinyl alcohol) interpenetrating polymer network type superporous hydrogels were prepared using a gas foaming method employing glyoxal as the crosslinking agent for Rosiglitazone maleate. Sodium bicarbonate was applied as a foaming agent to introduce the porous structure. Swelling behaviors of superporous hydrogel in acidic solution were studied to investigate their applications for gastric retention device. The optimum preparation condition of superporous hydrogels was obtained from the gelation kinetics. FT-IR, scanning electron microscopy, porosity and swelling ratio studies were used to characterize these polymers. *In vitro* drug release studies were also carried out.

**Results:**

The introduction of a small amount of Poly(Vinyl Alcohol) enhanced the mechanical strength but slightly reduced the swelling ratio. The prepared superporous hydrogels were highly sensitive to pH of swelling media, and showed reversible swelling and de-swelling behaviors maintaining their mechanical stability. The degradation kinetics in simulated gastric fluid showed that it had biodegradability. Swelling was dependent on the amount of chitosan and crosslinker. The drug release from superporous hydrogels was sustained for 6 hrs.

**Major Conclusion:**

The studies showed that chitosan-based superporous hydrogels could be used as a gastroretentive drug delivery system for rosiglitazone maleate in view of their swelling and prolonged drug release characteristics in acidic pH.

## INTRODUCTION

The principal aim of an oral controlled release drug delivery system is to achieve better bioavailability and release of the drug from the system, in a predictable and reproducible manner. A number of controlled drug delivery systems have been developed to prolong and control the release of drugs for a period of times in order to enhance their curing efficiencies (1). The drugs for oral delivery have their own convenience for easy and economic administration, but the problem is the loss of their functions due to the short residence in the body. About 80% of the administered drugs are excreted without being absorbed (2, 3). Many attempts have been made to prolong the residence time of drugs in the body for complete absorption, but not many systems have been successfully applied in practice. Various approaches to improve gastric residence include dosage forms like mucoadhesive or bioadhesive systems (4), high density systems (5), magnetic systems (6), superporous hydrogels (7), raft forming systems (8), low density systems (9) and floating ion exchange resins (10). Among these, superporous hydrogel system has been a promising one.

Hydrogels are crosslinked hydrophilic polymers with a network structure consisting of acidic, basic, or neutral monomers which are able to absorb large amounts of water. The swelling properties of hydrogels are mainly related to the elasticity of the network, the presence of hydrophilic functional groups (such as -OH, -COOH, -CONH_2_, -SO_3_H) in the polymer chains, the extent of crosslinking, and porosity of the polymer. A variety of stimuli sensitive hydrogels have been studied, but in many cases, slow response to environmental stimuli limited their effective uses (11). Although such slow swelling is beneficial for many applications, there are many situations where a fast swelling polymer is more desirable. Therefore, a new generation of hydrogels, which swell and absorb water very rapidly, has been developed. Examples of this new generation are SPH, which swell to an equilibrium size in a short period of time (12).

A superporous hydrogel (SPH) is a three-dimensional network of a hydrophilic polymer that absorbs a large amount of water in a very short period of time due to the presence of interconnected microscopic pores. When applied as drug carriers, these highly swollen hydrogels remain in stomach for a long time, releasing almost all loaded drugs, since their volumes are too big to transport through the pylorus and their sheer bulk hinder their transport to the next organ via the narrow pylorus. This unique swelling property allows them to be used as gastric retention carriers providing a sustained release through long residence in the stomach. In order to be used as an effective gastric retention device, the hydrogels are required to possess not only fast swelling but also following properties: biocompatibility, biodegradability, high swelling capacity, high mechanical strength, and stability in acidic condition (13).

Chitosan, a natural polysaccharide, is a biocompatible, biodegradable, and nontoxic material. Because chitosan has abundant amine groups within polymer chain, it dissolves in acidic solution and forms a gel with dialdehydes such as glutaraldehyde and glyoxal. Thus, in the low pH solution, chitosan hydrogels swell due to the presence of the positive charges in the network (14). Poly(vinyl alcohol) (PVA) is a well known hydrophilic, biocompatible, and commercially available polymer.

Rosiglitazone Maleate, an anti-diabetic agent, improves glycaemic control by increasing insulin sensitivity. It is a highly selective and potent agonist for the peroxisome proliferator-activated receptor- gamma (PPARγ) (15). Due to its short biological half-life (3.5±0.5 hrs) and instability at higher pH rosiglitazone maleate requires controlled release and was used as the drug in this study.

The interpenetrating polymer network of chitosan superporous hydrogel that was strengthened by PVA was prepared using a freezedrying/gas blowing technique and glyoxal as a crosslinking agent. The applications of the superporous hydrogels prepared in gastric retention devices were investigated by measurement of their swelling and mechanical properties. Emphasis was made upon the high swelling capacities and mechanical properties with a focus on stimuli-sensitive swelling and water-retention capacities which were highly demanded when the polymers are developed as a potential drug delivery system.

## MATERIAL AND METHODS

### 

#### Materials

Rosiglitazone maleate was obtained as a gift sample from Dr.Reddy's Labs, Hyderabad. Chitosan was purchased from Fluka (St. Gallen, Switzerland). Sodium bicarbonate (Loba Chemie, Mumbai), Glyoxal (40% water solution, Aldrich, Steinheim, Germany) and PVA (Sigma, Steinheim, Germany) were used in this study. All other reagents were of analytical grade.

#### Methods

##### Synthesis of superporous hydrogels

A 2% w/w stock solution was prepared by dissolving chitosan in 0.1M acetic acid. A 10% w/w aqueous PVA solution was also prepared. The chitosan and PVA solutions were mixed in the way that to have different compositions. Each chitosan/PVA mixture was placed in a test tube (inner diameter of 16 mm and height of 100 mm) and its pH value was adjusted to 5.0 by addition of acetic acid. A glyoxal aqueous solution, 10% w/w, was added to each chitosan/ PVA mixture. To the stock solution, was added 80 mg of sodium bicarbonate powder and the mixture was stirred vigorously to induce the gelation and foaming reactions, simultaneously. The foamed hydrogels were left to stand overnight at room temperature. The hydrogels were frozen in a deep freeze-drier at −60°C for 12 hrs. After freeze-drying, the samples were removed from the freeze-drier and thawed at room temperature during 4 hrs. Seven formulations were prepared by changing the amount of the ingredients as shown in table 1, (16).

**Table 1:** Formulations of chitosan superporous hydrogels.

Ingredients	I	II	III	IV	V	VI	VII
Chitosan (% w/w)	2	4	6	8	4	4	4
Poly Vinyl Alcohol (% w/w)	4	4	4	4	4	4	4
Glyoxal (% w/w)	4	4	4	4	2	6	8
Sodium Bicarbonate	80 mg	80 mg	80 mg	80 mg	80 mg	80 mg	80 mg
Rosiglitazone maleate	20 mg	20 mg	20 mg	20 mg	20 mg	20 mg	20 mg

##### Measurement of gelation kinetics

As the polymerization reaction proceeded, the viscosity continuously increased until the full network structure (gel structure) was formed. The gelation time was defined as a period of time for gel formation following addition of glyoxal and measured by a simple tilting method after adjustment of pH to 5.0 with acetic acid. It was determined by the duration of time taken by the reactant mixture to become viscous and the viscous solution no longer descended in the tilted tube position (17).

##### Drug loading

The method of soaking or equilibration was employed for drug loading. In this method the amount of buffer necessary for complete swelling of superporous hydrogel was determined. Thereafter the drug solution in the determined amount of buffer which was required for complete swelling was prepared. Subsequently, superporous hydrogel was placed in the drug solution and left until all the drug solution was sucked up. Then the completely swollen superporous hydrogel loaded with the drug was placed in an oven at 30°C for drying overnight (18).

##### Swelling studies

A completely dry, pre-weighed, disc-shaped superporous hydrogel was weighed and then immersed in excess of swelling medium. At various time intervals, the hydrogel was removed from the solution and weighed after excessive solution on the surface was blotted. Data presented in this experiment were the mean values of triplicate measurements. Results were calculated according to the following equation:

$$\text{Q}=\left({\text{M}}_{\text{s}}-{\text{M}}_{\text{d}}\right)/{\text{M}}_{\text{d}}$$

where *Q* is the swelling ratio, Ms the mass in the swollen state and M the mass in the dried state.

Five NaCl solutions (pH=1.2) with different ionic strengths (0.0001–1 M) were used to evaluate the salt effect on the swelling of superporous hydrogels. To study the pH sensitivity of the superporous hydrogels, HCl or NaOH solutions of the pH of 1.2, 2.0, 3.0, 4.9, 6.2 and 7.4 were used as swelling medium.

Pulsatile pH-dependent swelling of the superporous hydrogels was evaluated at 37°C by alternation of the swelling medium between the HCl solution (pH 1.2) and phosphate buffered solution (PBS, pH 7.4). The hydrogels were first swollen in pH 1.2 HCl solution for 30 min. The swollen hydrogels in the HCl solution were weighed at each given time and transferred to the PBS buffer. The same procedures were performed for swelling in PBS before transferring the swollen hydrogels back to the HCl solution. The hydrogels were transferred to the alternating solutions every 30 min (19).

##### Porosity measurement

For porosity measurement, the solvent replacement method was used. Dried hydrogels were immersed overnight in absolute ethanol and weighed after excess ethanol on the surface was blotted. The porosity was calculated from the following equation:

$$\text{Porosity}=({\text{M}}_{\text{2}}-{\text{M}}_{\text{1}}/\rho \text{V})$$

where M_1_ and M_2_ are the mass of the hydrogel before and after immersion in absolute ethanol, respectively; ρ is the density of absolute ethanol and V is the volume of the hydrogel (20).

##### Determination of void fraction

The void fraction was calculated by the following equation:

Void Fraction=Dimensional volume of the hydrogel/Total volume of pores

The void fraction inside superporous hydrogels was determined by immersing the hydrogels in HCl solution (pH 1.2) up to equilibrium swelling. The dimensions of the swollen hydrogels were measured and by using these data, sample volumes were determined as the dimensional volume. In the meantime, the amount of absorbed buffer into the hydrogels was determined by subtracting the weight of dried hydrogel from the weight of swollen hydrogel and the resulting values were assigned as the total volume of pores in the hydrogels (21).

##### Water retention

The following equation was used to determine the water retention capacity (WR_t_) as a function of time:

$$\text{W}{\text{R}}_{\text{t}}=\left({\text{W}}_{\text{p}}-{\text{W}}_{\text{d}}\right)/\left({\text{W}}_{\text{s}}-{\text{W}}_{\text{d}}\right)$$

where W_d_ is the weight of the dried hydrogel, W_s_ is the weight of the fully swollen hydrogel, and W is the weight of the hydrogel at various exposure times. For determination of the water-retention capacity of the hydrogels as a function of the time of exposure at 37°C, the water loss of the fully swollen polymer at timed intervals was determined by gravimetry (22).

##### Mechanical Properties

The compressive strengths of various superporous hydrogel formulations were determined using a bench comparator. Briefly, after the fully swollen hydrogel was put longitudinally under the lower touch of a bench comparator, different scale loads were successively applied on the upper touch until the point where the hydrogel could not support any more weight and completely fractured. The pressure at this point was defined as penetration pressure (PP) and calculated by the following equation:

$$\text{PP}={\text{F}}_{\text{u}}/\text{S}$$

where *F*_*u*_ is the ultimate compressive force at complete breakage of polymer and *S* is the contact area of the lower touch (23).

##### Determination of drug content

A weight of superporous hydrogel containing 4 mg of drug in 100 ml volumetric flask was treated with about 10 ml hydrochloric acid solution of pH 1.2 mixed well and made up to volume. The mixture was filtered and drug content was determined using UV-Vis spectrophotometer at 228 nm.

##### FT-IR spectroscopy

FT-IR spectroscopy was employed to ascertain the compatibility between the drug and the polymers. It was also used to investigate the chemical structure of the synthesized hydrogels. The FTIR spectrum was recorded over the range of 400–4000 cm^−1^ using KBr pellet method by Fourier-Transform Infrared (FT-IR) spectrophotometer, (Shimadzu, FT- IR 8400S, Japan).

##### Scanning electron microscopy

The dried superporous hydrogels were used for scanning electron microscopy (SEM) studies to determine the morphology of the dried samples. A JEOL JSM-840 scanning electron microscope (Jeol USA, Inc., Peabody, MA) was used after coating the samples with gold using a Hummer Sputter Coater (Technics, Ltd.). Images were captured using a digital capture card and Digital Scan Generator 1 (JEOL).

##### In vitro release studies

*In vitro* drug release of rosiglitazone maleate from the superporous hydrogels was evaluated in triplicates at 37±0.5 0C using a United States Pharmacopoeia (USP) Dissolution Test Apparatus Type 2 (paddle method) at a rotation speed of 50 rpm in 900 ml of 0.1M HCl (pH 1.2) for 6 hrs. At regular time intervals, 10 ml of the dissolution medium were withdrawn, replaced with an equivalent volume of fresh dissolution fluid and analyzed for the drug using a UV-Vis spectrophotometer (UV-1700, Shimadzu, Japan) at 228 nm (24). The obtained data were fitted into various release models. To determine release mechanism, the parameters n and k of the Korsmeyer-Peppas equation were computed (25).

## RESULTS AND DISCUSSION

### 

#### Synthesis of superporous hydrogels

**Figure 1:**
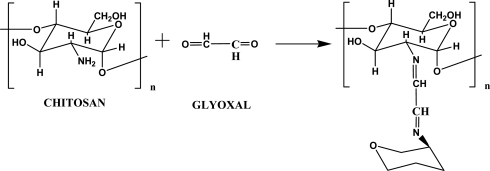
Synthesis of chitosan superporous hydrogel

In this study, chitosan and PVA were the polymers glyoxal was used as crosslinking agent and sodium bicarbonate was used as a porogen or foam generator and chitosan was crosslinked by glyoxal by condensation reaction (Schiff base reaction). PVA was crosslinked by physical method (i.e. weak bonding through a nonpermanent “association” of the polymer chains) employing freezing/thawing cycles due to direct hydrogen bonding, direct crystallite formation and liquid-liquid phase separation followed by a gelation mechanism. Crosslinking of chitosan and PVA, led to the formation of interpenetrating polymer network. Figure 1 shows the synthesis of chitosan superporous hydrogel using glyoxal (26).

#### Gelation kinetics

The gelation kinetics gives good information to determinine the introduction time of blowing agent (sodium bicarbonate). The most salient property of a superporous hydrogel is its fast swelling ability, because of the presence of large and uniform pores within the polymer structure which are produced due to the formation of foam at the time of polymerization. In order to produce large and uniform pores, the blowing agent must be introduced when the reactants have appropriate viscosity. Bubbles cannot maintain their shape for a long time if a gas blowing agent is added too early, or if gelation time is relatively longer. On the other hand, bubbles cannot even be formed if porogen is introduced too late or the gelation time is extremely short because, the reaction system becomes viscous at such a short period that the added porogen cannot produce bubbles. The foaming reaction took place only under the acidic condition (pH 5.0–5.5) and therefore the pH was adjusted to 5.0. The optimal pH for the gelation was around 7–8, where the polymerization proceeds rapidly and the gelling usually started within 0.5–1.0 min. Hence sodium bicarbonate was introduced 30 seconds after the addition of glyoxal.

#### Effect of the pH on the swelling capacity

**Figure 2:**
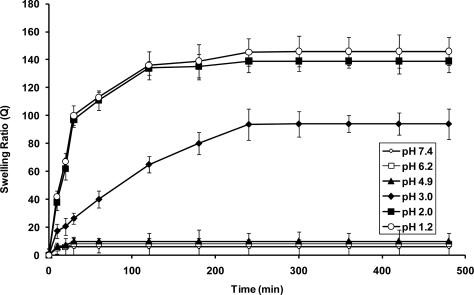
Effect of the pH of swelling medium on the swelling kinetics of the superporous hydrogel formulation IV (n=3, mean±standard deviation).

Swelling of the superporous hydrogels was reduced as the pH increased. Since the superporous hydrogels were composed of amine groups, which can dissociate or protonate at some suitable pH of the swelling media, the degree of swelling of superporous hydrogel underwent appreciable changes with external pH. Figure 2 shows the dynamic uptake of water of formulation IV in the solutions of pH 1.2, 2.0, 3.0, 4.9, 6.2, and 7.4. In acidic environment, chitosan superporous hydrogels showed higher swelling ratio compared to the basic environment since the amine groups in the chitosan molecules are ionized to ammonium ion (NH_3_^+^) in acidic aqueous media and these cationic charges in gel phase act as cationic repulsive forces between polymer molecules. Moreover, the ionization also caused an increase in ion osmotic pressure. These two factors and the capillary wetting of interconnected open pores of superporous hydrogel were thus responsible for a higher degree of swelling in the acidic pH. At higher pH values protonation of the amine groups led to decreased swelling ratios (14).

#### Effect of crosslinking density and chitosan on swelling capacity

**Figure 3:**
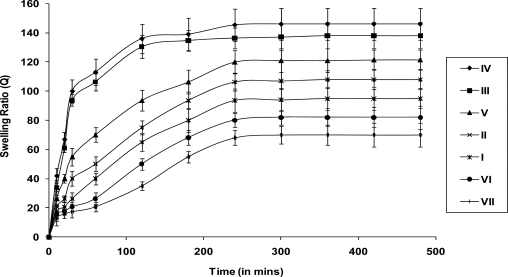
Swelling studies of superporous hydrogel formulations at pH 1.2 of HCl solution (n=3, mean±standard deviation).

The swelling ratios of all formulations in HCl solution of pH 1.2 is represented in figure 3. The swelling ratio of the prepared formulations in HCl solution was found to increase with time. The swelling ratios of superporous hydrogels decreased by increase in the crosslinking density, as much tighter networks were formed at higher concentration of crosslinking agents. The amount of crosslinking agent had influence on the swelling ratio of the polymer and as its concentration increased, polymer chains were attached to each other more strongly and the size of pores during foam formation was smaller. Furthermore, decrease in the chain flexibility reduced the swelling capacity of the polymer. Higher amount of chitosan resulted in higher swelling capacity as chitosan swells in acidic pH (16).

#### Effect of the ionic strength on the swelling capacity

**Figure 4:**
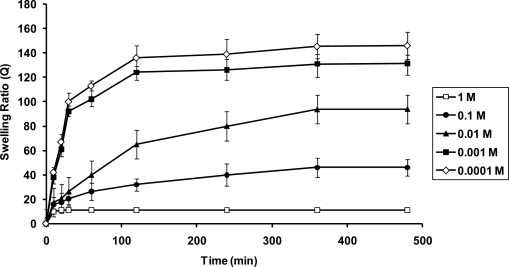
Effect of the ionic strength of swelling medium on the swelling kinetics of formulation IV (n=3, mean±standard deviation).

The effect of the ionic strength on the swelling capacity of formulation IV in figure 4 shows that an increase in the ionic strength within the range of 0.0001–1 M yields a significant decrease in the swelling ratio of formulation IV. When the ionic strength was less than 0.001 M, it did not affect the swelling behavior of superporous hydrogel. The sensitive swelling of the formulation towards ionic strength was attributed to the change in the charge distribution on the surface of the gel network. As the concentration of anions in the swelling medium increased, a stronger ‘‘charge screening effect’’ of the additional anions was achieved, causing imperfect anion-anion electrostatic repulsion and a decrease in osmotic pressure difference between the polymer network and the external solution. Therefore, swelling of superporous hydrogel was decreased. When the ionic strength was lesser than 0.001 M, the solution lacked enough ions to have any impact upon the charge on the surface of the polymer chains, leading to an inappreciable influence on the swelling of the superporous hydrogel (27).

#### Swelling reversibility studies

**Figure 5:**
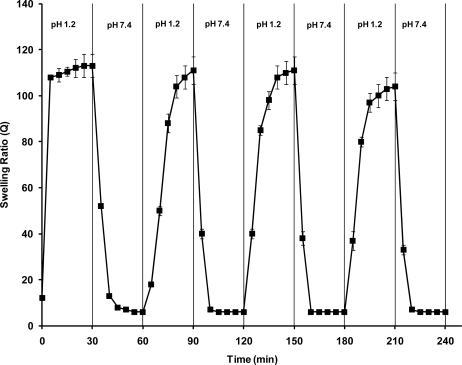
Pulsatile pH-dependent swelling behaviors of formulation IV at 37°C by alternation of the swelling medium between HCl solution (pH 1.2) and PBS (pH 7.4) (n=3, mean±standard deviation).

Figure 5 shows the swelling reversibility of the superporous hydrogel between pH 1.2 and PH 7.4 solutions. They were able absorb and deabsorb the swelling medium quickly upon the pH change from acidic to basic conditions quickly and vice versa. The structure of the superporous hydrogel with large numbers of pores connected to one another to form capillary channels was favorable for easy diffusion of the swelling medium into the polymeric matrix, thus contributing to its quick response toward pH change. The time for swelling was longer than that for deswelling of the hydrogels which might be due to the restricted chain mobility of the hydrogels which was anchored at several points through molecular entanglement with the PVA network, because the fast pH-sensitive behavior of hydrogels was based on the freely mobile side chains (28).

#### Porosity and void fraction measurements

As shown in table 2, the porosity of superporous hydrogels increased by the increase in amount of glyoxal. This is due to the incorporation of the higher crosslink density within the polymer structure leading to the decrease in the occupied volume. Additionally, the void fraction of the superporous hydrogels was decreased by the increase in amount of glyoxal which is consistent by the decrease in swelling ratio. The decrease in void volume led to a decrease in the amount of uptake of water into the structure, resulting in decrease in the swelling ratio (21, 22).

**Table 2:** Porosity, void fraction and penetration pressures of superporous hydrogel formulations (n=3, mean±standard deviation).

Formulations	Porosity (%)	Void fraction (ml/g)	Penetration pressure (g/cm^2^)
I	38.3±2.2	1.42±0.03	52±3
II	58.3±3.1	1.23±0.04	78±5
III	67.4±2.5	1.14±0.01	101±6
IV	73.2±4.2	0.93±0.03	126±8
V	46.2±3.3	1.31±0.02	61±3
VI	79.2±1.5	0.85±0.04	163±11
VII	88.2±2.1	0.72±0.03	202±12

#### Mechanical Properties

Appropriate mechanical strength should be provided to superporous hydrogels for their effective applications. Table 2 shows the penetration pressures of the formulations. The results indicate that increase in amount of crosslinking agent increased the penetration pressure values thus increasing the mechanical stability. The presence of PVA increased the overall crosslinking density of the superporous hydrogels by the polymer chains with PVA fibers. This entanglement significantly improved the structural integrity of the hydrogel and decreased stress relaxation, which enhanced its ability to withstand pressure (23).

#### Water retention

**Figure 6:**
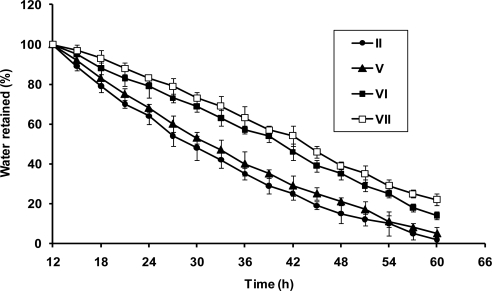
Water retention capacity of various formulations (n=3, mean±standard deviation). WR_t_ is expressed as the percentage of water retained in the polymer at certain time intervals.

As shown in figure 6, the weight loss of chitosan hydrogels occurred after 12 hrs. Lower the concentration of the crosslinking agent, the faster was the loss of water from the superporous hydrogel. In acidic environment, superporous hydrogels kept equilibrium swelling ratios for a certain period of time, being protonated as ammonium ions. D-glucosidic linkages in chitosan were slowly cleaved by acid hydrolysis. As amine groups stabilize D-glucosidic linkage's cleavage by acids, a part of chitosan oligomers, especially not highly crosslinked, were slowly dissolved in the swelling media, inducing the weight loss of samples. The interconnected pores allowed the polymer to hold more water by capillary force. The superporous hydrogel consisting of higher amount of glyoxal decreased polymer rigidity, thus improving the resiliency of the polymer in response to compression and prevention of the water loss efficiently. Hence an increase in the amount of glyoxal decreased the rate of loss of water (14).

#### Determination of drug content

The drug content analysis showed that the drug loading is uniform it is distributed in the superporous hydrogels. Properly and the drug content was in the range of 96.5–98.6% of the total amount of the drug added.

#### Chemical identification

**Figure 7:**
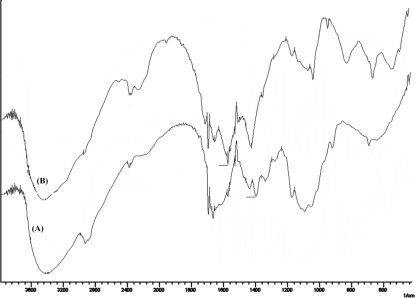
FTIR spectra of chitosan (A) before and (B) after gelation (crosslinking).

The chemical structure of the synthesized superporous hydrogel was identified by FTIR where amino peak of the chitosan was observed at 1402 cm^−1^ (Figure 7) and the imine bond (C=N) produced from the crosslinking between the chitosan amino group and the glyoxal aldehyde group via a Schiff base reaction was observed at 1572 cm^−1^ (29).

#### Drug - excipient compatibility

**Figure 8:**
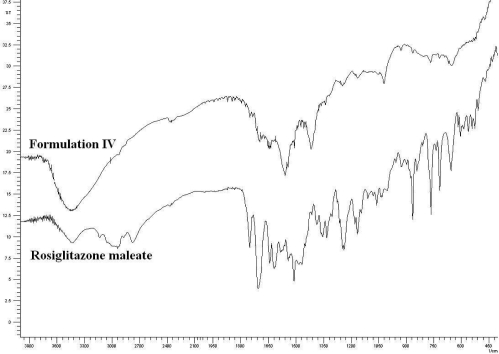
FT-IR Spectra of Rosiglitazone maleate and its superporous hydrogel formulation IV.

The position of peaks in FT-IR spectra of pure drug was compared with those in FT-IR spectra of drug with the superporous hydrogel (Figure 8) for this purpose. The broad peak at 3408 cm^−1^ in the spectra of pure drug corresponding to -OH group, the peaks at 1690 cm^−1^ and 1740 cm^−1^ indicating the presence of carbonyl group, the peak at 1620 cm^−1^ shows the presence of pyridine structure. The peak at 630 cm^−1^ corresponding to C=S bonding and no disappearance or significant shift in the peak position of drug in any spectra of drug with superporous hydrogel proved that the drug and polymers used for the study are compatible (30).

#### Structure and size of pores

**Figure 9:**
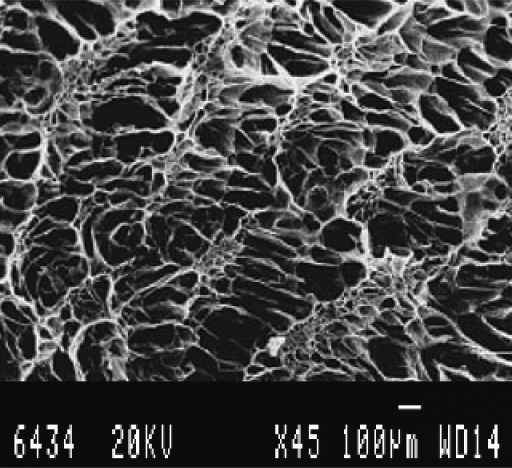
Scanning electron microscopic photograph of formulation IV recorded at 45X magnification showing porous surface.

The scanning electron microscopic photograph of superporous hydrogel (Figure 9) clearly shows the presence of pores on the surface. The superporous hydrogel has high porosity and is responsible for faster swelling of superporous hydrogels when compared to conventional hydrogels.

#### In vitro release studies

**Figure 10:**
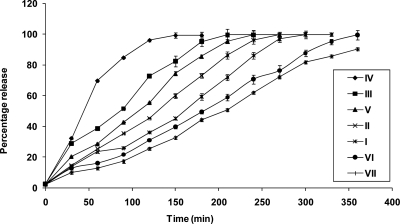
In vitro release profiles of Rosiglitazone maleate from the prepared formulations (n=3, mean±standard deviation).

The drug release profiles of the drug from the hydrogels are shown in figure 10. The release of the drug was found to be dependent to the amount of chitosan and crosslinker and as the amount of chitosan increased, the release was faster since chitosan is a pH sensitive monomer. Drug release was found to be inversely related to the amount of crosslinking agent and at higher crosslink density where the openings are less release is also low. The best fit model was found to be Korsmeyer-peppas model and since the value of n, the time exponent, calculated from this equation was found to be greater than 1 in all the drug release profiles, the release mechanism is assumed to be super case-II transport, wherein multiple release mechanisms exist (25).

## CONCLUSION

Suitable chitosan superporous hydrogels, which swelled and de-swelled reversibly depending on the pH of media, and having good mechanical properties, were successfully formulated. The high porosity of the superporous hydrogel is achieved by the carbon dioxide formation during the polymerization process, resulting in a capillary structure of interconnecting pores. The high polar internal surface of these pores within the superporous hydrogels was responsible for the very fast swelling rate which resulted in high swelling ratio of the superporous hydrogels. In acidic environment, superporous hydrogels showed higher swelling ratio because of the presence of amine groups as cations in acidic condition. The drug release profile may be easily related to the swelling properties of these superporous hydrogels. This study demonstrates that superporous hydrogels of chitosan may be suitable for use as a gastroretentive drug delivery system.
